# Investigation of Abdominoplasty Without General Anesthesia: A Scoping Review

**DOI:** 10.1177/22925503241301711

**Published:** 2024-12-18

**Authors:** Ted Zhou, Madeline E. Hubbard, Nasimul S. Huq

**Affiliations:** 1 Department of Medicine, 3710 McMaster University, Hamilton, ON, Canada; 2 Department of Surgery, 3710 McMaster University, Hamilton, ON, Canada

**Keywords:** abdominoplasty, anesthesia, plastic surgery, literature review, safety, abdominoplastie, anesthésie, chirurgie plastique innocuité

## Abstract

**Introduction:** Abdominoplasty is a common aesthetic surgical procedure primarily performed under general anesthesia (GA). However, GA is aerosol-generating and involves extended immobilization associated with systemic complications like venous thromboembolisms (VTEs). There is increasing interest in performing abdominoplasties without GA because of potential lower complication rates and shorter postoperative recovery time. This review sought to summarize all available literature on the safety and outcomes of abdominoplasty performed without GA. **Methods:** A scoping review was conducted with no date limits in October 2023 encompassing Medline, Embase, Web of Science, and CINAHL. The type of anesthesia was separated into 3 categories: conscious or intravenous (IV) sedation, regional anesthetic blocks (RAB: spinal and epidural), and local anesthesia (direct local infiltration and field blocks). **Results:** A total of 28 studies were included. Safety data was reported for abdominoplasty alone (*n* = 6), with liposuction (*n* = 14), or both (*n* = 1). The employed anesthesia methods were IV and local (*n* = 13), RAB and local (*n* = 3), IV and RAB (*n* = 2), IV and RAB and local (*n* = 2), and IV only (*n* = 1). A total of 48 379 patients were identified, with 30 cases of VTEs reported. Two studies reported GA conversion rates between 4.8% and 6.0%. A total of 11 studies assessed abdominoplasty outcomes, highlighting high patient satisfaction and low postoperative pain. The majority of analyzed studies had a “high” or “critical” risk of bias. **Conclusion:** Our review provides preliminary evidence that performing abdominoplasty without GA is safe and feasible. Additional high-quality studies are necessary to further validate our findings and to develop a standardized approach.

## Résumé

**Introduction :** L’abdominoplastie est une procédure chirurgicale esthétique courante pratiquée principalement sous anesthésie générale (AG). Cependant, l’AG génère des aérosols et implique une immobilisation prolongée associée à des complications systémiques, telles que la maladie veineuse thromboembolique (MVTE). Un intérêt croissant se fait jour pour la réalisation d’abdominoplasties sans AG en raison des taux moindres de complications potentielles et d’une durée de convalescence postopératoire plus courte. Cette revue a cherché à résumer toutes les publications disponibles sur la sécurité et les résultats de l’abdominoplastie pratiquée sans AG. **Méthodes :** Une revue d’ensemble a été menée sans limites de date en octobre 2023 dans les bases de données Medline, Embase, Web of Science et CINAHL. Les types d’anesthésie ont été séparés en trois catégories : sédation consciente ou intraveineuse (IV), blocs anesthésiques régionaux (BAR : médullaires et périduraux) et anesthésie locale (infiltration locale directe, blocs de champs). **Résultats :** Un total de 28 études ont été incluses. Les données d’innocuité ont été rapportées pour la seule abdominoplastie (n = 6), avec liposuccion (n = 14) ou les deux (n = 1). Les méthodes employées étaient les suivantes : IV et locale (n = 13), BAR et locale (n = 3), IV et BAR (n = 2), IV et BAR et locale (n = 2) et IV seule (n = 1). Un total de 48 379 patients ont été identifiés, avec 30 cas de MVTE rapportés. Deux études ont rapporté des taux de conversion de l’AG entre 4,8 et 6,0%. Un total de 11 études ont évalué les résultats de l’abdominoplastie, soulignant le haut niveau de satisfaction des patients et une faible douleur postopératoire. La majorité des études analysées avaient un risque de biais « élevé » ou « critique ». **Conclusion :** Notre revue fournit des données probantes préliminaires attestant que la réalisation d’une abdominoplastie sans AG est sécuritaire et faisable. Des études supplémentaires de qualité élevée sont nécessaires pour valider et élaborer davantage une approche standardisée.

## Introduction

Abdominoplasty is the sixth most commonly performed aesthetic surgical procedure in the world, with the main objective being to reshape the abdominal region by excising redundant skin and fat tissue.^1^ Abdominoplasty is typically performed under general anesthesia (GA) with endotracheal intubation.^2^ This is an aerosol-generating medical procedure, which requires additional personal protective measures in the context of the COVID-19 pandemic.^3^

Surgery under GA leads to long periods of immobilization, which increases the risk of serious systemic complications, such as deep vein thrombosis (DVT) or pulmonary embolism (PE), collectively known as venous thromboembolism (VTE). Previously reported incidences of VTE arising from abdominoplasty range from 0.3% to 2%.^1,4^ The risk of VTEs increases for patients who undergo abdominoplasty with additional procedures, are over 40 years of age, and have risk factors such as prior VTEs or hypercoagulable states.^5^ Moreover, VTE prophylaxis is a controversial issue, with varying recommendations on preoperative actions that can be taken to reduce risk.^6^

There has been increasing interest in employing local and/or regional anesthetic for surgical procedures. These techniques, such as tumescent solution and epidural blocks, have been linked to potential advantages, such as lower reported incidence of postoperative complications and earlier ambulation time for patients.^7^ Furthermore, local and regional anesthetics can be performed in outpatient settings and are nonaerosol-generating procedures, increasing flexibility for the surgeon. To date, there has been no literature review investigating the safety profile and outcomes of abdominoplasty under conscious sedation, regional anesthesia, and/or local anesthesia.

To better inform clinical practice, we conducted a scoping review of all available literature reporting on the safety and postoperative outcomes of abdominoplasty performed without GA.

For safety, our primary objective was to synthesize data regarding the incidence rate of VTEs. Our secondary objective was to synthesize other relevant adverse event data, including local surgical site complications, unplanned hospitalizations, and the rate of conversion of the procedure to GA. For postoperative outcomes, our primary objective was to summarize postoperative pain scores and patient satisfaction.

## Methods

This scoping review was conducted following the PRISMA-ScR protocol for scoping reviews.^8^ The PRISMA Extension for Scoping Reviews was used as the reporting guideline for the scoping review manuscript. The protocol has been previously registered on Open Science Framework (OSF: https://osf.io/n5s49).

### Eligibility Criteria

Inclusion criteria consisted of (1) any published studies that reported on abdominoplasty procedures with or without liposuction; (2) surgery was conducted under any anesthesia method other than GA. Anesthesia methods included conscious or intravenous sedation, regional anesthetic blocks (RAB: spinals and epidurals), or local anesthesia. Local anesthesia was deemed to be either direct local infiltration of anesthetic (including nerve blocks) or a field block. Any type of study design was included.

Studies were excluded if they (1) did not report on abdominoplasty outcomes or safety; (2) were not published in English; (3) were an abstract or conference proceeding; (4) only reported on postoperative anesthesia use.

### Information Sources and Search Strategy

A search strategy was developed in conjunction with a health sciences librarian. The databases searched were MEDLINE, Embase, Web of Science, and CINAHL. The search strategy was based on 2 main concepts: abdominoplasty and non-GA. Relevant subject headings linked to the keywords were included, and the search was conducted on October 19, 2023. A sample search strategy for MEDLINE is provided in Supplemental Table S1.

### Study Screening

Two independent reviewers conducted title, abstract, and full-text screening in-duplicate. Screening conflicts were discussed until a consensus was reached. A third reviewer acted as a third opinion if screening discrepancies could not be resolved.

### Data Extraction

Data from eligible studies were extracted to an Excel spreadsheet by 2 independent reviewers in-duplicate. Then, both reviewers met to come to a consensus on the extracted data. Any discrepancies were resolved through discussion with a third reviewer. The original data extraction sheet can be found on OSF (https://osf.io/ubveq/?view_only = 90e8f38b828342b2b64ece43a289317d).

### Risk of Bias in Individual Studies

The evaluation of the risk of bias was conducted using “ROBINS-I” for nonrandomized studies and “RoB 2.0” for randomized trials.^9,10^ The outlined tools were employed by 2 separate reviewers (TZ and MH), and a consensus was reached for each domain and for the overall risk of bias. Any disagreements were resolved by a third reviewer (NH). Case reports and series were not evaluated given their inherent high risk of bias.

### Synthesis of Results

Descriptive and quantitative analyses were performed when reporting on safety and postoperative outcomes. Safety was primarily defined as the incidence rates of VTEs. Safety also included local surgical site complications, conversion to GA, and unplanned hospitalizations. For postoperative outcomes, we focused on pain scores and patient satisfaction. Tabular methods were used to display study results.

## Results

**Figure 1.**
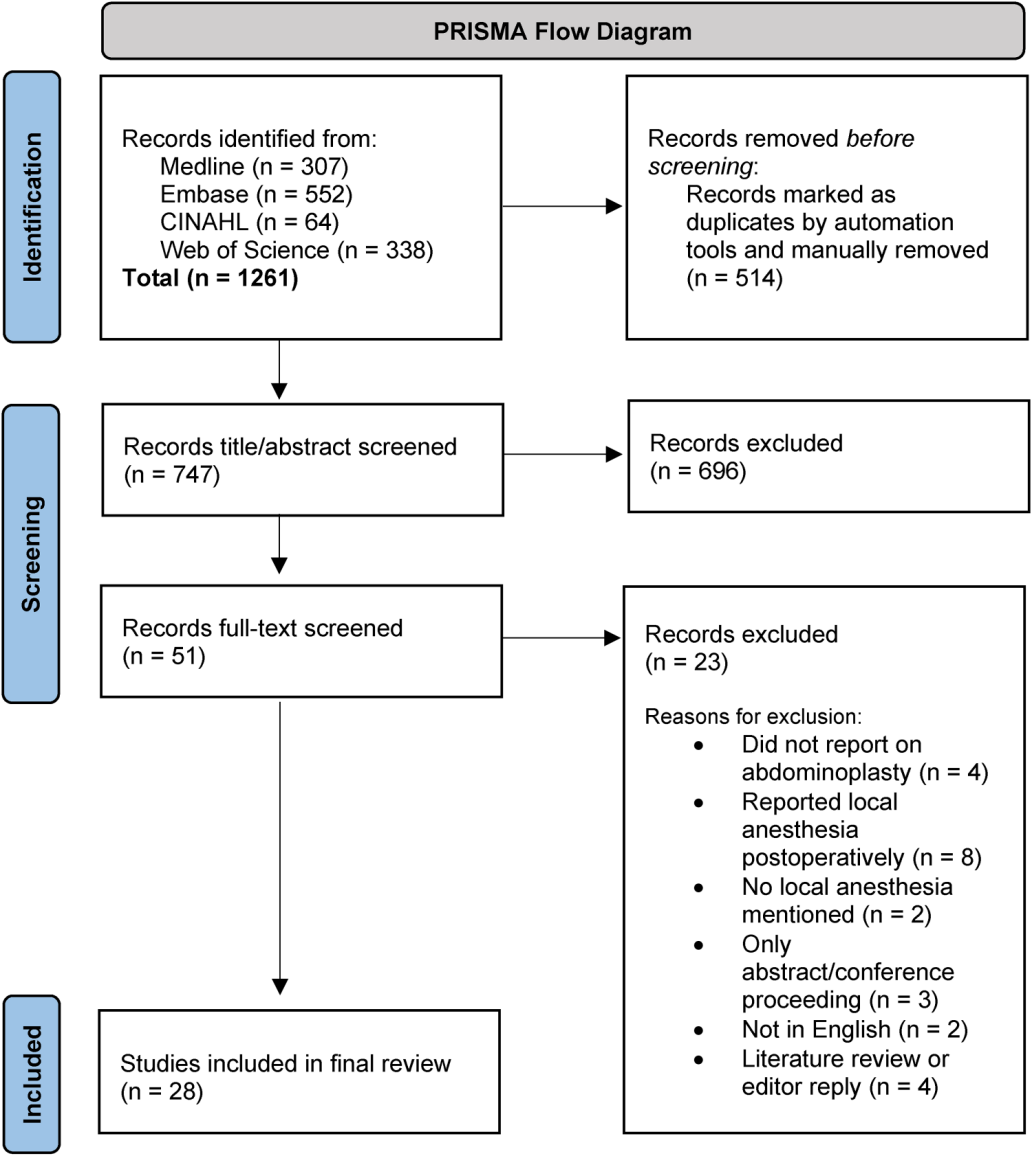
Preferred reporting items for systematic reviews and meta-analyses (PRISMA) flow diagram.

A PRISMA flow diagram outlining study identification and screening is shown in Figure 1. A total of 28 studies satisfied the inclusion criteria. Of these studies, 5 were case reports/series,^11–15^ 18 were retrospective cohort/chart studies,^7,16–32^ 2 were prospective cohort studies,^2,33^ 1 was a randomized prospective feasibility study,^34^ 1 was a survey,^35^ and 1 was a methods paper.^36^ The publication dates ranged from 1979 to 2021, with only 5 studies published in the last ten years (2014-2023).^2,13,24,33,34^ All patients undergoing abdominoplasty without GA were either ASA Class I or II. Studies employed a combination of monitored intravenous (IV) sedation, RAB, and local anesthesia.

### Safety Data for Abdominoplasty Without Liposuction

A total of 6 studies reported on abdominoplasty safety outcomes without liposuction.^12,18,19,21,27,29^ The anesthesia methods were IV sedation and local (*n* = 5, 83.3%),^18,19,21,27,29^ and a combination of IV sedation, RAB and local (*n* = 1, 16.7%).^12^ In the 6 studies, a total of 634 patients were included. There were no incidences of any VTEs or other serious adverse events. A total of 45 local complications (7.1%) were reported, with the most common adverse events being seromas (*n* = 29, 4.6%), local minor tissue necrosis (*n* = 7, 1.1%), and wound dehiscence (*n* = 5, 0.8%). Only one study (*n* = 145 participants) reported on nonsurgical site-related adverse events, which were: postoperative nausea (*n* = 6, 4.1%), urine retention (*n* = 1, 0.7%), and hematuria (*n* = 1, 0.7%).^21^ There were no reported conversions to GA, and all patients were discharged the same-day after surgery. Only one patient was hospitalized overnight after surgery, based on patient request for nonsurgical-related reasons.^19^ See Table 1 for a summary of safety data.

**Table 1.** Safety Data for Abdominoplasty Without General Anesthesia.

**Study**	**Patient Characteristics**	**Anesthesia Used**			**Safety**			
		CS/IV	RAB	Local	Local Complications	Systemic/Other Complications	GA Conversion	Hospital Stay
Brauman and Capocci, 2009	n = 337, Age: 36, BMI: 29.3	Y	N	Y	5 seromas, 6 local minor tissue necrosis	None	None	None
Byun et al, 1999	n = 20, Age: 41.7	Y	N	Y	None	None	None	n = 1
Failey et al, 2013	n = 145, Age: 40.3, BMI: 25.7	Y	N	Y	19 seromas, 5 wound dehiscence, 2 suture granulomas, 2 hematomas	6 postoperative nausea, 1 urine retention, 1 hematuria	None	None
Kodeih et al, 2009	n = 1, Age: 46	Y	Y	Y	None	None	None	None
Mottura, 1993	n = 25	Y	N	Y	1 local minor tissue necrosis	None	None	None
Rosenberg et al, 2001	n = 106, Age: 45	Y	N	Y	5 seromas	None	None	None
**Total Studies: *n* = 6**	***n* = 634**	***n* = 6**	***n* = 1**	***n* = 6**	**Seromas: *n* = 29, Local Tissue Necrosis: *n* = 7, Wound Dehiscence: *n* = 5, Other: *n* = 4**	**Postoperative Nausea: *n* = 6, Urine Retention: *n* = 1, Hematuria: *n* = 1**	**n = 0**	**Hospital Stay: *n* = 1**

Abbreviations: CS, conscious sedation; IV, intravenous; RAB, regional anesthetic block (epidural and spinal); GA, general anesthesia.

Age and BMI data are presented as mean values unless otherwise indicated.

### Safety Data for Abdominoplasty With Liposuction

A total of 14 studies reported on abdominoplasty safety outcomes with liposuction.^7,13–16,22,25,28,31–34^ The anesthesia methods were IV sedation and local (*n* = 8, 57.1%),^2,7,14,25,28,31–33^ RAB and local (*n* = 3, 21.4%),^16,22,34^ IV sedation and RAB (*n* = 2, 14.3%),^11,15^ and all 3 techniques (*n* = 1, 7.1%).^13^

One study outlined the feasibility and safety of performing abdominoplasties under IV sedation and local anesthesia by presenting data related to over 3000 cases performed by the senior author, but did not specify the number of patients.^7^ The study reported no cases of VTEs, occasional postoperative nausea and vomiting, and a seroma rate of < 5%.

In the remaining 13 studies that reported on abdominoplasty with liposuction, a total of 1046 patients were included. There were no reported incidences of VTEs. There were 42 cases of local complications, with the most common adverse events being seromas (*n* = 22, 4.0%), scar revisions (*n* = 6, 0.6%), and surgical site infections (*n* = 4, 0.4%). In 2 of these studies, there were 24 patients who were converted to GA, with an incidence rate ranging from 4.8% to 6%, respectively.^22,34^ There were no overnight hospitalizations reported. Two studies (*n* = 160 participants) reported on nonsurgical site-related adverse events, which were: intraoperative nausea (*n* = 25, 15.6%), intraoperative vomiting (*n* = 13, 12.4%), postoperative nausea or vomiting (*n* = 11, 10.5%), and urinary retention (*n* = 15, 14.3%).^2,34^ See Table 2 for a summary of safety data.

**Table 2.** Safety Data for Abdominoplasty and Liposuction Without General Anesthesia.

		**Anesthesia Used**			**Safety**			
**Study**	**Patient Characteristics**	CS/IV	RAB	Local	Local Complications	Systemic/Other Complications	GA Conversion	Hospital Stay
Abramson, 1998	n = 6	Y	N	Y	2 seromas	None	None	None
Brauman et al, 2018	n = 256, Age: 44.1, BMI: 29.4	Y	N	Y	10 seromas, 6 scar revisions, 1 hematoma, 1 surgical site infection.	None	None	None
Burns and Meland, 2007	n = 1, Age: 50, BMI: 25.3	Y	Y	N	None	None	None	None
Hafezi and Nouhi, 2005	n = 4, Age: 42	N	Y	Y	None	None	None	None
Hafezi et al, 2011	n = 371	N	Y	Y	None	None	*n* = 18 (4.8%)	None
Kryger et al, 2004	n = 153	Y	N	Y	10 seromas, 3 surgical site infections, 2 skin necrosis, 1 abscess, 1 pseudobursa excision	None	None	None
Leal et al, 2021	n = 1, Age: 35	Y	Y	Y	None	None	None	None
Metry et al, 2019	n = 100	N	Y	Y	None	25 intraoperative nausea, 13 intraoperative vomiting, 2 postoperative nausea and vomiting, 15 urinary retention	*n* = 6 (6%)	None
Mohamed et al, 2018	n = 60, Age: 33.3, BMI: 31.7	Y	N	Y	None	9 postoperative nausea and vomiting	None	None
Nguyen et al, 1997	n = 35, Age: 42	Y	N	Y	None	None	None	None
Rudkin et al, 2008	n = 2, Age: 44.5, BMI: 25.5	Y	Y	N	None	None	Not reported	Not reported
Wilkinson and Swartz, 1985	n = 35	Y	N	Y	1 skin necrosis, 2 disruption of internal sutures, 2 skin folding of the lower abdomen	None	Not reported	Not reported
Williams et al, 2005	n = 22, Age: 40, BMI: 27	Y	N	Y	None	None	None	None
Mustoe et al, 2007	n = Unspecified “more than 3000”	Y	N	Y	Unspecified scar revisions, localized infections at incision site, and seroma	Unspecified postoperative nausea and vomiting	None	None
**Total Studies: *n* = 14**	**n = 1046**	***n* = 11**	***n* = 6**	***n* = 12**	**Seroma: *n* = 22, Scar Revision: *n* = 6, Surgical Site Infection: *n* = 4, Skin Necrosis: *n* = 3, Other: *n* = 7**	**Intraoperative Nausea/Vomit: *n* = 38, Postoperative Nausea/Vomit: *n* = 11, Urinary Retention: *n* = 15**	**GA Conversion: *n* = 24**	**Hospital Stay: *n* = 0**

Abbreviations: CS, conscious sedation; IV, intravenous; RAB, regional anesthetic block (epidural and spinal); GA, general anesthesia.

Age and BMI data are presented as mean values unless otherwise indicated.

### Safety Data for Abdominoplasty With and Without Liposuction

One study combined safety data for patients receiving abdominoplasty with and without liposuction. Keyes et al investigated the incidence and predictors of VTEs in patients undergoing abdominoplasty with GA compared to IV sedation.^24^ A total of 235 274 abdominoplasties under GA and 46 699 cases under IV sedation were analyzed. A total of 201 patients (0.09%) undergoing GA and 30 patients (0.06%) undergoing IV sedation experienced a VTE. The study found no increased VTE risk by type of anesthesia.

### Safety Data for Cosmetic Surgeries Performed Without General Anesthesia Including Abdominoplasty

In 5 studies, the authors presented safety information for various cosmetic surgeries conducted without GA, including abdominoplasty. However, the studies did not provide specific details on adverse events solely related to abdominoplasty.

Blake published a study reviewing 4800 patients who had undergone cosmetic surgical procedures performed in an office setting.^17^ The anesthetic technique was IV sedation using propofol and ketamine, combined with a local anesthetic injection with intercostal nerve blocks in the cases of abdominoplasty. There were no reported incidences of VTEs, GA conversions, or hospitalizations.

Michaels and Eko^26^ reported on 68 patients who underwent abdominoplasty, 29 of which were performed with a rib block and IV sedation. There were no reported incidences of VTEs, GA conversions, or hospitalizations.

Shestak^30^ reported on 29 total abdominoplasties, 17 of which were performed with IV sedation and local anesthesia. There were no reported incidences of VTEs, GA conversions, or hospitalizations.

Ersek reviewed over 30 000 cosmetic surgical procedures performed under IV sedation and local anesthetic, including abdominoplasty.^20^ There were no reported incidences of VTEs, GA conversions, or hospitalizations.

Hasen et al^23^ reported on 169 patients receiving aesthetic procedures, including abdominoplasty, performed under IV sedation with either midazolam/fentanyl or propofol. There were no reported incidences of VTEs, GA conversions, or hospitalizations.

### Other

Lewis^36^ was a methods paper detailing a procedure labeled “midabdominoplasty,” which requires only local anesthetic. The paper reported that the study author had not yet experienced a complication at the time of publication.

Matarasso et al^35^ was a survey investigating the types of anesthesia used by plastic surgeons while performing limited and full abdominoplasties. The study found that of the limited abdominoplasties performed (*n* = 2003), 11% were done under IV sedation administered by an anesthesiologist, 5% were done by IV sedation administered by the surgeon, and 1% were performed under local anesthesia alone. Of the 11 016 full abdominoplasties performed, 7% used IV sedation administered by an anesthesiologist, and 2% used IV sedation administered by the surgeon.

### Abdominoplasty Outcomes

Of the 28 studies, a total of 11 studies reported abdominoplasty outcomes by either assessing patient satisfaction (*n* = 8),^2,18,19,28,29,32–34^ pain (*n* = 2),^15,26^ or both (*n* = 1).^23^

Of the 8 studies that reported only on patient satisfaction, 5 collected data with either an unspecified satisfaction survey or an unspecified method.^18,28,29,32,33^ Three of these studies reported that their patients had “high levels” of satisfaction.^18,32,33^ One study stated that all patients were satisfied with their results, as aesthetics were within patients’ range of expectations.^28^ Lastly, one study stated that patients were generally pleased with the results of surgery.^29^

The remaining 3 studies outlined their patient satisfaction assessment method. Byun et al^19^ administered a satisfaction survey with a 50% response rate (*n* = 10 participants), showing that all participants returned to normal activities after abdominoplasty under IV sedation within 3 weeks, and that all participants stated that they would be willing to undergo other procedures under IV sedation in the future. Mohamed et al^2^ surveyed their patients (*n* = 60) using a 5-point satisfaction scale. They found that 40% of patients were highly satisfied, 30% were satisfied and would request it again if required, 26.7% had good satisfaction and may request it again if required, and 3.3% had bad satisfaction and would not undergo the procedure again. Lastly, Metry et al^34^ compared 2 groups of patients undergoing abdominoplasty, either with GA or a spinal block. The study used an 11-point satisfaction scale, finding that the satisfaction score was higher in the spinal block group but with no statistical significance.

Two studies reported postoperative pain outcomes after surgery. Michaels and Eko^26^ compared 2 groups undergoing abdominoplasty, one with GA, and the other with IV sedation and a rib block. An 11-point visual analogue scale (VAS) for pain was employed, showing that the rib block group had statistically significantly less postoperative pain than the GA counterpart. Lastly, Rudkin et al^15^ used an 11-point scale to show that both their patients that underwent abdominoplasty with a paravertebral block did not exceed 4/10 pain severity, at either rest or with movement.

One study assessed both postoperative pain and patient satisfaction. Hasen et al^23^ compared 2 groups of patients that underwent aesthetic surgeries using IV sedation with either midazolam/fentanyl or propofol alone. The study used an 11-point numerical rating scale for pain, finding no difference between groups. A satisfaction survey was also administered, revealing that 91% to 96% of participants would choose to undergo IV sedation instead of GA for similar procedures in the future.

### Risk of Bias Assessment

The results of the risk of bias assessment for included studies can be found in Tables 3 and 4. All nonrandomized studies demonstrated a “serious” or “critical” risk of bias. The one included a randomized controlled trial demonstrated “some” risk of bias. Overall, the risk of bias in analyzed studies is high.

**Table 3.** Risk of Bias Assessment for Included Nonrandomized Studies (ROBINS-I).

**Study**	**D1**	**D2**	**D3**	**D4**	**D5**	**D6**	**D7**	**Overall**
Wilkinson et al, 1985	Serious	Critical	Serious	Serious	Moderate	Serious	Moderate	**Critical**
Mottura, 1993	Serious	Critical	Moderate	Serious	Low	Serious	Moderate	**Critical**
Nguyen et al, 1997	Serious	Low	Moderate	Moderate	Low	Serious	Moderate	**Serious**
Shestak et al, 1998	Serious	Low	Moderate	Low	Low	Serious	Moderate	**Serious**
Byun et al, 1999	Serious	Low	Low	Serious	Moderate	Moderate	Moderate	**Serious**
Rosenberg et al, 2001	Serious	Low	Moderate	Serious	Low	Serious	Moderate	**Serious**
Ersek, 2003	Serious	Serious	Low	Serious	Serious	Moderate	Moderate	**Serious**
Hasen et al, 2003	Serious	Low	Low	Serious	Low	Moderate	Serious	**Serious**
Kryger et al, 2004	Serious	Low	Low	Serious	Low	Moderate	Moderate	**Serious**
Williams et al, 2005	Serious	Low	Low	Moderate	Low	Serious	Moderate	**Serious**
Hafezi and Nouhi, 2005	Serious	Low	Low	Serious	Low	Moderate	Moderate	**Serious**
Mustoe et al, 2007	Serious	Critical	Moderate	Serious	Low	Serious	Moderate	**Critical**
Blake, 2008	Serious	Serious	Moderate	Serious	Low	Low	Moderate	**Serious**
Brauman and Capocci, 2009	Serious	Low	Low	Serious	Low	Moderate	Moderate	**Serious**
Michaels and Eko, 2009	Serious	Low	Low	Moderate	Low	Moderate	Moderate	**Serious**
Hafezi et al, 2011	Serious	Moderate	Low	Serious	Serious	Moderate	Moderate	**Serious**
Failey et al, 2013	Serious	Low	Low	Serious	Low	Moderate	Moderate	**Serious**
Brauman et al, 2018	Serious	Low	Low	Serious	Low	Moderate	Moderate	**Serious**
Keyes et al, 2018	Serious	Low	Low	Serious	Low	Moderate	Serious	**Serious**
Mohamed et al, 2018	Serious	Moderate	Low	Moderate	Low	Moderate	Moderate	**Serious**

D1, bias due to confounding; D2, bias in selection of participants into the study; D3, bias in classification of interventions; D4, bias due to deviations from intended interventions; D5, bias due to missing data; D6, bias in measurement of outcomes; D7, bias in selection of the reported result.

**Table 4.** Risk of Bias Assessment for Included Randomized Controlled Trials (RoB 2.0).

**Study**	**D1**	**D2**	**D3**	**D4**	**D5**	**Overall**
Metry et al, 2019	Low	Low	Low	Some	Some	**Some**

D1, bias arising from randomization process; D2, bias due to deviations from the intended interventions; D3, bias due to missing outcome data; D4, bias in measurement of the outcome; D5, bias in selection of the reported result.

## Discussion

Our review highlights preliminary evidence that suggests abdominoplasty without GA is safe and feasible. A total of 21 studies reported on abdominoplasty-specific safety data with and without liposuction for 48 379 patients. Of these cases, there were 30 reported cases of VTEs, and no hospitalizations due to excessive pain. There was a combined VTE incidence rate of 0.06%. This is lower than VTE incidence rates of abdominoplasty with GA reported by other reviews (0.3%-2%).^1,4^ The 11 studies that reported on postoperative pain and/or patient satisfaction showed that abdominoplasty with GA was well-tolerated, and that patients had a favorable opinion of surgeries performed without GA.

The low VTE incidence rate reported when performing abdominoplasty without GA parallels findings from other surgeries. Previous studies investigating procedures such as facelifts, blepharoplasties, and aesthetic breast surgeries performed without GA have demonstrated favorable safety profiles, with low VTE incidence rates, shorter operative times, and faster times to ambulation for patients.^37–39^ For example, a survey of VTE incidence rates following facelift surgery found that procedures performed under local anesthesia had shorter operative times and a lower VTE rate when compared to facelifts performed under GA.^40^ Earlier ambulation and shorter length of surgery are both factors associated with decreased thrombosis risk, which may help to explain possible lower VTE incidence when surgeries are performed without GA.^41^ Although the existing literature on the benefits of performing plastic surgery procedures without GA appear promising, data from other surgical specialties remains conflicting. Studies investigating procedures performed by orthopedic and general surgeons without GA have shown mixed data on whether surgeries done under local and/or regional anesthesia improve the safety profile.^42–44^ Our review highlights the safety of abdominoplasty without GA, demonstrated by a low overall VTE incidence rate (0.06%). However, it is difficult to delineate whether performing abdominoplasty without GA has an inferior, equivalent, or superior safety profile to abdominoplasty with GA due to the lack of comparative studies. Further research is required to validate our findings and to compare the safety profile of abdominoplasty under different anesthetic modalities.

In the 11 studies that reported on postoperative pain scores and patient satisfaction, abdominoplasty without GA was well-tolerated. Results reported by the studies generally showed high satisfaction with minimal postoperative pain. This finding bolsters existing evidence that plastic surgery procedures performed without GA have favorable patient satisfaction and an acceptable pain profile. Stahl et al performed aesthetic breast surgeries under sedation and local anesthetic, finding that the majority of patients were satisfied with their anesthesia and had well-controlled pain.^45^ However, the methodology used for assessing postoperative outcomes among studies included in our review remains lacking. The majority of outcome data was not assessed using validated outcome measurement tools, and there was a paucity of information on outcome assessment methodology.^46^ Only 44.4% (n = 4/9) of studies reporting on patient satisfaction specified the assessment method, and none of these studies employed validated outcome assessment tools. This increases the risk of bias and makes it difficult to synthesize postoperative outcome data between studies. To improve the quality of assessment methodology, the use of patient-reported outcome measures, such as the BODY-Q, should be employed when assessing abdominoplasty to ensure the collection of accurate, comprehensive data on clinically meaningful outcomes from a patient's perspective.^47^ Our review suggests that abdominoplasty without GA is well-tolerated by patients and is feasible to perform. There is still a lack of robust data to comprehensively evaluate the procedure from a patient perspective and to compare outcomes between studies due to high heterogeneity in assessment methodology.

All included nonrandomized studies were found to be at “high” or “serious” risk of bias. Of the 28 studies analyzed by our review, there was only one randomized controlled feasibility study comparing abdominoplasty under GA to RAB and IV sedation.^34^ This highlights a paucity of high-quality, prospective studies to draw conclusions from. Currently, available evidence suggests that abdominoplasty without GA is safe and has favorable postoperative outcomes. However, surgeons should consider that there remains minimal high-quality evidence to guide clinical practice when performing abdominoplasty without GA.

Our review highlights 3 key research areas requiring further improvement: (1) the lack of comparative studies on postoperative outcomes and safety for abdominoplasty with versus without GA; (2) the limited use of validated outcome assessment tools; and (3) the paucity of high-quality studies available to establish evidence-based recommendations. Future studies on abdominoplasty without GA should focus on addressing these gaps to improve the quality of research produced, to better inform practicing surgeons, and to optimize outcomes for patients undergoing abdominoplasty without GA.

### Limitations

The lack of high-quality studies limited the quality of conclusions that could be drawn. There was high heterogeneity in postoperative outcome assessment methods, limiting the ability to synthesize data.

## Conclusions

This review provides preliminary evidence that performing abdominoplasty without GA is safe, feasible, and well-tolerated by patients. Our review stresses the need for additional, well-designed comparative studies to further validate our findings. There is currently a paucity of high-quality evidence to base recommendations, and surgeons should employ their judgment and expertise when performing abdominoplasty without general anesthesia.

## Supplemental Material

sj-docx-1-psg-10.1177_22925503241301711 - Supplemental material for Investigation of Abdominoplasty Without General Anesthesia: A Scoping Review Supplemental material, sj-docx-1-psg-10.1177_22925503241301711 for Investigation of Abdominoplasty Without General Anesthesia: A Scoping Review by Ted Zhou, Madeline E. Hubbard and Nasimul S. Huq in Plastic Surgery

sj-docx-2-psg-10.1177_22925503241301711 - Supplemental material for Investigation of Abdominoplasty Without General Anesthesia: A Scoping Review Supplemental material, sj-docx-2-psg-10.1177_22925503241301711 for Investigation of Abdominoplasty Without General Anesthesia: A Scoping Review by Ted Zhou, Madeline E. Hubbard and Nasimul S. Huq in Plastic Surgery
