# Supplementary material for: Investigation of Abdominoplasty Without General Anesthesia: A Scoping Review
Source: Plast Surg (Oakv). 2024 Dec 18;34(1):94–103. doi: 10.1177/22925503241301711 (PMC11653385; doi:10.1177/22925503241301711)
Supplement: sj-docx-1-psg-10.1177_22925503241301711 - Supplemental material for Investigation of Abdominoplasty Without General Anesthesia: A Scoping Review [file sj-docx-1-psg-10.1177_22925503241301711.docx]

**Supplementary Table S1.** Search Strategy for Medline

| **Concept** | **Search Terms** |
| --- | --- |
| Abdominoplasty | 1. Tummy tuck OR abdominoplast OR apronectom* 2. exp Abdominoplasty/​ 3. 1 OR 2 |
| Anesthesia | 1. anesthe* OR epidural OR spinal OR nerve block OR local OR lidocaine OR bupivacaine OR ropivacaine OR tumescen* OR “conscious sedation” 2. exp Anesthesia/ or exp anesthesia, Epidural/, exp Anesthesia, Local/ or exp Anesthesia, Spinal/, exp Anesthsia, Intravenous/ 3. 4 OR 5 |
| **Result** | 1. **3 AND 6 (n = 307)** |
